# Modeling of Charge-to-Breakdown with an Electron Trapping Model for Analysis of Thermal Gate Oxide Failure Mechanism in SiC Power MOSFETs

**DOI:** 10.3390/ma17071455

**Published:** 2024-03-22

**Authors:** Jiashu Qian, Limeng Shi, Michael Jin, Monikuntala Bhattacharya, Atsushi Shimbori, Hengyu Yu, Shiva Houshmand, Marvin H. White, Anant K. Agarwal

**Affiliations:** 1 Department of Electrical & Computer Engineering, The Ohio State University, Columbus, OH 43210, USA; shi.1564@osu.edu (L.S.); jin.845@osu.edu (M.J.); bhattacharya.119@osu.edu (M.B.); yu.3868@osu.edu (H.Y.); houshmand.3@osu.edu (S.H.); white.1829@osu.edu (M.H.W.); 2 Ford Motor Co., Dearborn, MI 48126, USA; ashimbor@ford.com

**Keywords:** thermal gate oxide, SiC, MOSFET, charge-driven breakdown, *Q_BD_*, CCS, CVS, PVS, electron trapping model

## Abstract

The failure mechanism of thermal gate oxide in silicon carbide (SiC) power metal oxide semiconductor field effect transistors (MOSFETs), whether it is field-driven breakdown or charge-driven breakdown, has always been a controversial topic. Previous studies have demonstrated that the failure time of thermally grown silicon dioxide (SiO_2_) on SiC stressed with a constant voltage is indicated as charge driven rather than field driven through the observation of Weibull Slope β. Considering the importance of the accurate failure mechanism for the thermal gate oxide lifetime prediction model of time-dependent dielectric breakdown (TDDB), charge-driven breakdown needs to be further fundamentally justified. In this work, the charge-to-breakdown ($Q_{BD}$) of the thermal gate oxide in a type of commercial planar SiC power MOSFETs, under the constant current stress (CCS), constant voltage stress (CVS), and pulsed voltage stress (PVS) are extracted, respectively. A mathematical electron trapping model in thermal SiO_2_ grown on single crystal silicon (Si) under CCS, which was proposed by M. Liang et al., is proven to work equally well with thermal SiO_2_ grown on SiC and used to deduce the $Q_{BD}$ model of the device under test (DUT). Compared with the $Q_{BD}$ obtained under the three stress conditions, the charge-driven breakdown mechanism is validated in the thermal gate oxide of SiC power MOSFETs.

## 1. Introduction

Silicon carbide (SiC) power MOSFETs are gradually gaining market attention due to their lower switching losses, higher temperature capability, higher switching frequencies, and increasingly competitive price compared to their silicon (Si) counterparts [1,2]. Especially in the field of electric vehicles (EVs), the aforementioned advantages make them largely attractive to EV OEMs and tier-one suppliers for potential applications in onboard chargers and drivetrain inverters [3,4,5]. Planar SiC power MOSFETs, with their relatively more mature process and cheaper manufacturing costs, have become the mainstream commercial SiC power MOSFETs on the market [6,7,8,9,10,11]. Trench SiC power MOSFETs, although optimized in device performance due to enhanced electron mobility and elimination of JFET resistance, as well as smaller cell pitch, still hold a relatively small market share due to their higher cost and lower reliability [12,13]. The lower reliability is mainly caused by electric field crowding at the corner of the trench gate and implantation-induced basal plane dislocation (BPD) [14,15,16,17]. Therefore, although the performance and structural limitations of planar SiC power MOSFETs are gradually becoming apparent, unless trench SiC power MOSFETs with better economy and reliability are commercialized, the main way to improve the performance of planar SiC power MOSFETs is to operate the devices at a higher gate oxide field to increase the channel electron density [18,19]. However, this places more stringent demands on the reliability of gate oxide in planar SiC power MOSFETs. One major area of concern is that the prediction of gate oxide lifetime under the typical operation, with an increased gate oxide field, still needs to meet conservative design requirements [20]. This requires not only improvements to the thermal growth process of gate oxide in planar SiC power MOSFETs, but also sufficient accuracy in the gate oxide lifetime prediction model [21]. The key to determining the accuracy of the prediction model is the failure mechanism of thermal gate oxide grown on SiC [22].

The commonly used gate oxide lifetime prediction method in the industry for planar SiC power MOSFETs is the time-dependent dielectric breakdown (TDDB) test. As MOSFETs are voltage-controlled devices, the conventional TDDB test in the industry is constant voltage stress TDDB (CVS-TDDB) based on the thermochemical E model, as it provides the most conservative lifetime extrapolation, even without physical or even experimental justification [23,24,25]. The thermochemical E model is considered to be based on the thermal gate oxide failure mechanism of field-driven breakdown [26]. P. Moens et al. questioned this mechanism and proposed a more rational failure mechanism of charge-driven breakdown [27]. The team grew approximately 53 nm of silicon dioxide (SiO_2_) on n-epi SiC to form circular capacitor structures with n+ doped polysilicon gates. By measuring the gate leakage current as a function of the gate oxide field at different temperatures, they concluded that the electron tunneling mechanism from SiC to SiO_2_ transitions from thermally assisted tunneling (TAT) to Fowler–Nordheim tunneling (FNT) as the gate oxide field increases. During the transition, there is a phase where both the electron tunneling mechanisms jointly influence. This transition in the electron tunneling mechanism cannot be described in the conventional Weibull plots of CVS-TDDB based on field-driven breakdown but can be accurately depicted in new Weibull plots based on charge-driven breakdown, where the Weibull Slope (β) at the TAT dominant stage, FNT dominant stage, and the joint influence stage each have a specific value that decreases in the stage order of TAT dominance, joint influence, and FNT dominance. Therefore, the failure mechanism of thermal gate oxide grown on SiC is considered to be charge-driven breakdown rather than field-driven breakdown, and a more optimistic lifetime prediction model based on $Q_{BD}$ has been proposed. Since the stressor is charge rather than field, constant current stress (CCS) is considered as a better stress method because it is not negatively affected by trapped electrons in the gate oxide and can reach $Q_{BD}$ faster [28]. The β value of the Weibull plots based on the CCS-$Q_{BD}$ approach has also been proven to accurately describe the transition in the electron tunneling mechanism.

This work draws on the electron trapping model in very thin SiO_2_ (no more than 10 nm) thermally grown on Si under CCS by M. Liang et al., proving its applicability also in approximately 4–5 times thicker SiO_2_ thermally grown on SiC through CCS-TDDB tests on thermal gate oxide in a type of commercial planar SiC power MOSFETs until failure [29]. Based on this electron trapping model, a $Q_{BD}$ model for thermal gate oxide in the commercial planar SiC power MOSFETs under test is established. Considering that the gate voltage signal for MOSFETs is a pulse-width-modulated (PWM) signal rather than a constant in actual applications, MOSFETs are voltage-controlled devices [30]. Therefore, in addition to conventional CVS, this paper also extracts the $Q_{BD}$ of the thermal gate oxide in commercial planar SiC power MOSFETs under pulsed voltage stress (PVS) and CCS at different stress levels for comparison with the established $Q_{BD}$ model. The high match between the extracted $Q_{BD}$ and the $Q_{BD}$ model indicates that different stress methods do not change the failure mechanism of thermal gate oxide, and the existence of a specific $Q_{BD}$ that causes the thermal gate oxide to fail under different stress methods further proves that charge-driven breakdown is the failure mechanism of thermal gate oxide. Additionally, the lifetime prediction model established based on this failure mechanism can be considered more credible even if it is not as conservative as the thermochemical E model [31]. This will also provide a theoretical basis for suggesting the industry adopt more aggressive screening methods to more effectively screen out extrinsic defects in thermal gate oxide according to the more optimistic lifetime prediction [32].

## 2. Materials and Methods

### 2.1. Devices under Test (DUTs)

**Figure 1:**
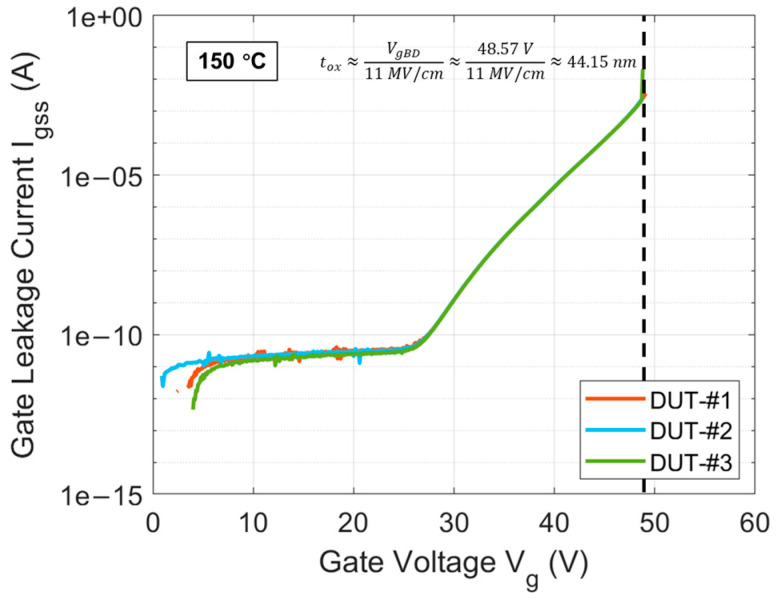
$I_{gss}$ curves as a function of $V_{g}$ at 150 °C until oxide breakdown for three DUTs. The dashed line indicates the oxide breakdown voltage.

In this work, the devices under test (DUTs) are commercial 1200 V planar SiC power MOSFETs packaged in TO-247-3 from Vendor E. Considering that as more and more EV OEMs upgrade from 400 V systems to 800–900 V systems, the voltage rating of 1200 V will receive more attention from the market, so selecting this DUT is a better reference for the industry [33,34,35,36,37]. The curves of gate leakage current ($I_{gss}$) for three DUTs at 150 °C, as a function of gate voltage ($V_{g}$), are presented in Figure 1. The high overlap of the three curves demonstrates the high uniformity in gate oxide quality of the commercial DUTs. This indicates that these commercial DUTs undergo stringent gate oxide screening before leaving the factory, reducing the adverse impact of early oxide failure caused by extrinsic defects on subsequent test results [38]. The $I_{gss}$ curves for all three DUTs exhibit breakdown near 50 V, with an average gate oxide breakdown voltage of about 48.57 V. Based on the assumption that the critical breakdown electric field is about 11 MV/cm for SiO_2_, the gate oxide thickness of DUTs can be estimated to be approximately 44.15 nm [39]. According to the total capacitance ($C_{tot}$) derived from the gate C-V measurements of DUTs and $C_{ox}$ of SiO_2_, the gate oxide area in each DUT is estimated to be about 0.9 mm^2^. General information of the commercial DUTs used in this work is summarized in Table 1.

### 2.2. Experimental Methods

#### 2.2.1. Liang and Hu’s Electron Trapping Model

A mathematical model for describing the electron trapping phenomenon in very thin SiO_2_ thermally grown on Si under CCS has been proposed by M. Liang and C. Hu [29]. In this model, M. Liang et al. have demonstrated that when the thickness of SiO_2_ in a polycrystalline-Si-SiO_2_-Si MOS capacitor structure reaches a certain level, the change in $V_{g}$ ($∆V_{g}$) between the polycrystalline-Si gate and the grounded Si measured under CCS tends to saturate at a high electron fluence (F). However, in the case of thinner SiO_2_, $∆V_{g}$ does not show a saturation trend with F but instead tends to linearly increase until the oxide breakdown. This phenomenon is also observed under various CCS, and with different thicknesses of SiO_2_, as long as they do not exceed the critical oxide thickness. Therefore, for thinner SiO_2_, M. Liang et al. believe that in addition to the pre-existing electron traps in SiO_2_, new electron traps are being generated during CCS. The pre-existing electron traps and the generated electron traps, having different trap capture cross-sections and trap centroids, collectively capture electrons tunneling from Si into the oxide, thus affecting $∆V_{g}$. Based on this, a comprehensive mathematical model is established and used to characterize the electron trapping phenomenon in the 100 Å SiO_2_ of a fabricated Si MOS capacitor structure.

In this model, the density of filled electron traps can be expressed as follows:

(1) $$N_{ot}\left(\sigma_{p},\sigma_{g},F\right)=N_{opt}\left(\sigma_{p},F\right)+N_{ogt}\left(\sigma_{g},F\right)=N_{op}\left(\sigma_{p}\right)\left(1-e^{-\sigma_{p}F}\right)+q\frac{g}{J}[F-\frac{1}{\sigma_{g}}(1-e^{-\sigma_{g}F})]$$

where

$N_{ot}$—density of filled electron traps;

$N_{op}/N_{opt}$—density of pre-existing total/filled electron traps;

$N_{ogt}$—density of filled generated electron traps;

$\sigma_{p}/\sigma_{g}$—capture cross-section of pre-existing/generated electron traps;

q—electric charge of an electron;

J—current density of the specific CCS;

F—electron fluence (F=J·t/q, t is the stress time under the specific CCS);

g—generation rate of generated electron traps under the specific CCS.

**Figure 2:**
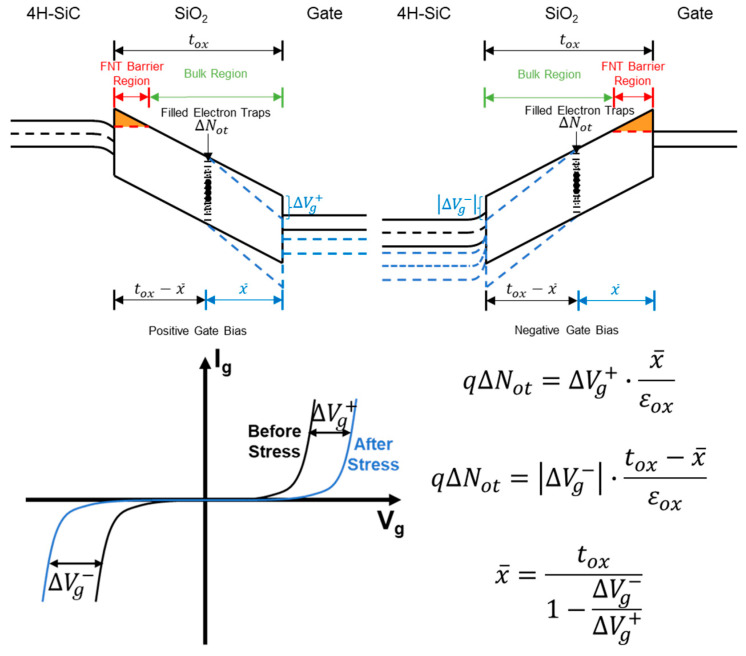
Energy band variation caused by $∆V_{g}$ to maintain a constant FNT barrier for a constant $\left|I_{gss}\right|$. $\bar{x}$ can be extracted based on $∆V_{g}$ at different stages under the constant $\left|I_{gss}\right|$.

Therefore, $∆V_{g}$ due to the filled electron traps can be expressed as follows:

(2) $$∆V_{g}\left(F\right)=\frac{q}{\varepsilon_{ox}}\bar{x}(F)N_{ot}\left(\sigma_{p},\sigma_{g},F\right)$$

where $\varepsilon_{ox}$ is the dielectric constant of SiO_2_ and $\bar{x}$ is the centroid of electron traps measured from the gate. Figure 2 presents a method for extracting $\bar{x}$ with respect to F through shifts in $I_{g}$-$V_{g}$ curves at different stages under a specific CCS as shown below.

Also, $\bar{x}$ can be represented by the centroid of pre-existing electron traps ($\bar{x_{p}}$) and the centroid of generated electron traps ($\bar{x_{g}}$) as follows:

(3) $$\bar{x}\left(F\right)=\frac{\bar{x_{p}}N_{opt}+{\bar{x_{g}}N}_{ogt}}{N_{opt}+N_{ogt}}$$

When F is large enough under a specific CCS, $∆V_{g}$ can be simplified to the following:

(4) $$∆V_{g}\left(F\right)=\frac{q}{\varepsilon_{ox}}\left[\bar{x_{p}}N_{op}-\bar{x_{g}}q\frac{g}{J}\frac{1}{\sigma_{g}}+\bar{x_{g}}q\frac{g}{J}F\right]=\frac{q}{\varepsilon_{ox}}\bar{x_{g}}q\frac{g}{J}\cdot F+\frac{q}{\varepsilon_{ox}}\left(\bar{x_{p}}N_{op}-\bar{x_{g}}q\frac{g}{J}\frac{1}{\sigma_{g}}\right)$$

Considering that $\bar{x_{p}}N_{op}$ is a constant characteristic value regarding pre-existing electron traps and the generation rate of generated electron traps g under a specific CCS is also considered as a specific constant value in the model, Equation (4) can be regarded as a linear expression of $∆V_{g}$ with respect to F when F is large enough. Moreover, differentiating Equation (4) can give the constant slope of this linear expression as follows:

(5) $$\frac{d∆V_{g}}{dF}=\frac{q}{\varepsilon_{ox}}\bar{x_{g}}q\frac{g}{J}$$

From Equation (3), it is known that $\bar{x}$ varies due to the ratio change between $N_{opt}$ and $N_{ogt}$ under different F. When F is large enough, $N_{opt}$, having tended to saturate earlier, becomes almost negligible relative to $N_{ogt}$, which continues to increase with the constant generation rate of new electron traps. In this case, $\bar{x}$ tends to saturate, and the saturation value approached can be estimated as $\bar{x_{g}}$. In the model, $\bar{x_{g}}$ is found to be a constant value, unaffected by CCS. This phenomenon is also reflected in the measurements of gate oxide in commercial SiC DUTs in this work.

#### 2.2.2. Extraction of Charge-to-Breakdown ($Q_{BD}$)

$Q_{BD}$ measurement is a standard destructive method used to determine the quality of gate oxide in MOS devices. $Q_{BD}$ is extracted by calculating the total charge passing through the dielectric (i.e., the product of total electron fluence and the electric charge of an electron, or the integral of electron current over time-to-breakdown ($t_{BD}$), making it a time-dependent measurement [27]. The extraction of $Q_{BD}$ can be represented as follows:

(6) $$Q_{BD}=q\cdot F\cdot A_{ox}=\int_{0}^{t_{BD}}I\left(t\right)d(t)$$

## 3. Results

### 3.1. Modeling of $∆V_{g}$ When Breakdown Occurs ($∆V_{gBD}$) in Commercial SiC DUTs

#### 3.1.1. $\bar{x_{g}}$ Extraction

**Figure 3:**
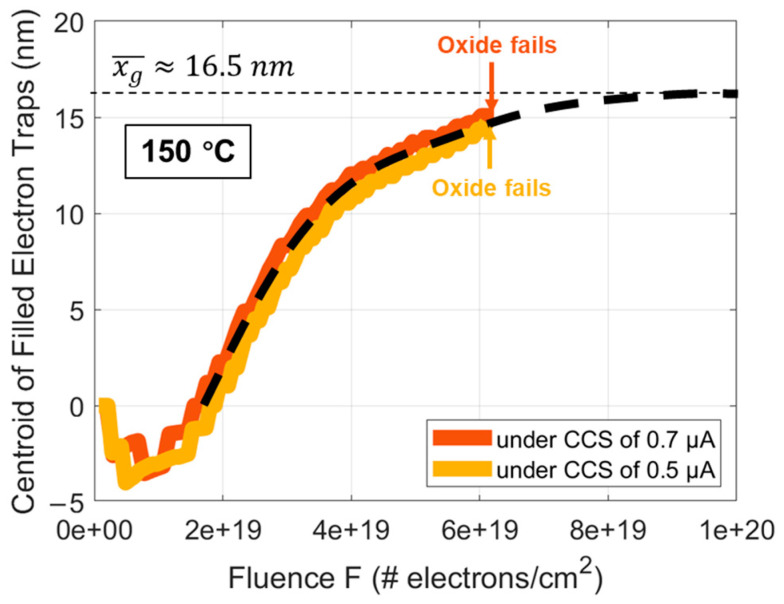
$\bar{x}$ curves as a function of F for DUTs at 150 °C under a CCS of 0.5 and 0.7 μA, respectively, using the extraction method introduced in Figure 2.

In Figure 3, based on the above method of extracting $\bar{x}$, the curves of $\bar{x}$ versus F for the commercial SiC DUTs at 150 °C under a CCS of 0.5 and 0.7 μA are shown. It is observable that the two curves highly coincide, consistent with what is measured in the oxide thermally grown on Si that there is no correlation with the CCS. However, due to the inferior quality of oxide thermally grown on SiC compared to Si, the oxide fails before F is large enough for $\bar{x}$ to reach its saturation value [40]. Therefore, by fitting the overlapped curves of $\bar{x}$ versus F, the $\bar{x_{g}}$ of DUTs is estimated to be approximately 16.5 nm measured from the gate.

#### 3.1.2. Mathematical Expression of $∆V_{gBD}$

**Figure 4:**
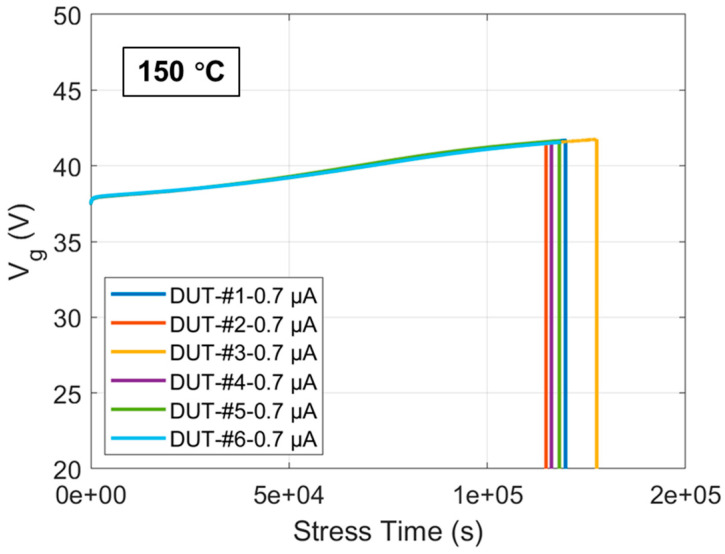
$V_{g}$ curves as a function of stress time until oxide breakdown at 150 °C for six DUTs under a CCS of 0.7 μA.

**Figure 5:**
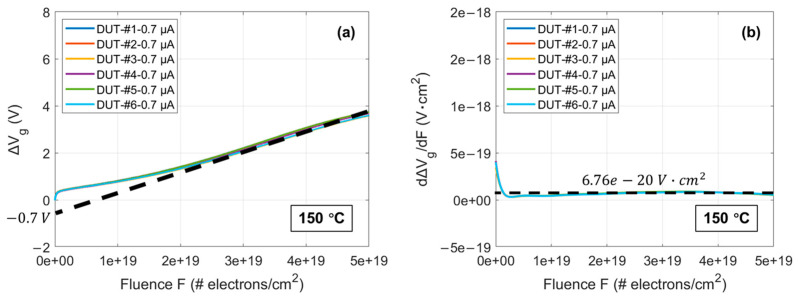
(**a**) $∆V_{g}$ curves as a function of F until oxide breakdown at 150 °C for the six DUTs under a CCS of 0.7 μA; (**b**) Differentiated curves from (**a**). # is a number sign representing the number of electrons.

In Figure 4, the curves of $V_{g}$ over stress time until the oxide breakdown at 150 °C for six DUTs under a CCS of 0.7 μA are shown. $∆V_{g}$ can be obtained by subtracting the initial $V_{g}$ from $V_{g}$ at different time points. Multiplying time by the known current density under CCS and dividing by the electric charge of an electron yields the electron fluence. Figure 5a presents the curves of $∆V_{g}$ versus F until the oxide breakdown at 150 °C for the six DUTs under a CCS of 0.7 μA. Differentiating the curves in Figure 5a results in the curves shown in Figure 5b. The high consistency among the six curves in both again proves the uniformity of the oxide quality in these commercial SiC DUTs after a possible stringent gate oxide screening. According to Equation (4), the electron trapping phenomenon in the oxide of these commercial SiC DUTs shows characteristics similar to those predicted by the model for very thin oxide thermally grown on Si. By extending the linear part of the curves within the high F range in Figure 5a to intersect with the y-axis, the value of intersection point is estimated to be approximately −0.7 V. Additionally, the saturation value extracted in Figure 5b within the corresponding F range for the linear part of the curves in Figure 5a is about 6.76 × 10^−20^ V·cm^2^. Therefore, the relevant mathematical expressions can be represented as follows:

(7) $$\frac{q}{\varepsilon_{ox}}\left(\bar{x_{p}}N_{op}-\bar{x_{g}}q\frac{g}{J}\frac{1}{\sigma_{g}}\right)\approx -0.7\;V$$

(8) $$\frac{q}{\varepsilon_{ox}}\bar{x_{g}}q\frac{g}{J}\approx \;6.76\;\times \;10^{-20}\;V\cdot {\mathrm{cm}}^{2}$$

Since J≈ 0.7 μA/0.9 mm^2^ ≈ 7.8 × 10^−5^ A/cm^2^ and $\bar{x_{g}}\approx$ 16.5 nm, the above expressions can be transformed into the following:

(9) $$g(0.7\;\mu A)\approx \;4.3\;\times \;10^{7}\;{\mathrm{cm}}^{-2}\cdot s^{-1}$$

(10) $$\bar{x_{p}}N_{op}-\frac{1.46\times 10^{-13}}{\sigma_{g}}\approx -1.51\times 10^{6}{\mathrm{cm}}^{-1}$$

**Figure 6:**
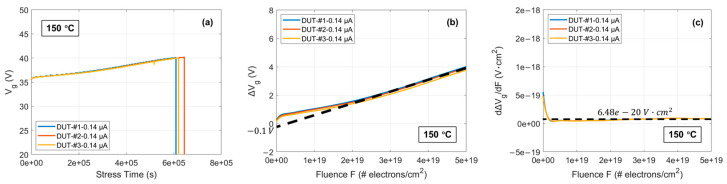
(**a**) $V_{g}$ curves as a function of stress time until the oxide breakdown at 150 °C for three DUTs under CCS of 0.14 μA; (**b**) $∆V_{g}$ curves as a function of F until the oxide breakdown at 150 °C for the three DUTs under CCS of 0.14 μA; (**c**) Differentiated curves from (**b**).

Similarly, Figure 6a displays the curves of $V_{g}$ over stress time until the oxide breakdown for three DUTs under CCS of 0.14 μA. Moreover, both the characteristics of $∆V_{g}$ versus F for three DUTs under a CCS of 0.14 μA shown in Figure 6b, and of the differentiated curves in Figure 6c, are very similar to those in Figure 5a,b. Therefore, by repeating the aforementioned method, similar relevant mathematical expressions can be obtained as

(11) $$g(0.14\;\mu A)\approx \;8.47\;\times \;10^{6}\;{\mathrm{cm}}^{-2}\cdot s^{-1}$$

(12) $$\bar{x_{p}}N_{op}-\frac{1.43\times 10^{-13}}{\sigma_{g}}\approx -2.157\times 10^{5}{\mathrm{cm}}^{-1}$$

Considering that the DUTs stressed under a CCS of 0.7 and 0.14 μA are from the same batch of identical devices produced on the same wafer using exactly the same process, $\bar{x_{p}}N_{op}$ can be considered a constant value unaffected by CCS. Also, in the model, the generated electron traps under CCS have been proven to have a centroid always at a specific and constant position unaffected by CCS, with CCS mainly affecting their generation rate. Furthermore, $\sigma_{g}$, as a specific attribute of the generated electron traps, is also considered to be a constant value unaffected by CCS. Therefore, relating Equations (10) and (12) can give $\bar{x_{p}}N_{op}$ and $\sigma_{g}$ values of approximately 6.2 × 10^7^ cm^−1^ and 2.317 × 10^−21^ cm^2^. Since the electron-fluence-to-breakdown ($F_{BD}$) with respect to $t_{BD}$ of the oxide can be expressed as $F_{BD}=J\cdot t_{BD}/q$, and with the CCS value I≈0.009·J, the mathematical relationship between $∆V_{gBD}$ and I can be expressed as follows:

(13) $$∆V_{gBD}\left(I\right)\approx \;7.7\;\times \;10^{-13}\;\cdot \;g\left(I\right)\cdot t_{BD}\;-\;4.68\;\times \;10^{-13}\;\cdot \;\frac{g\left(I\right)}{I}+\;28.74\;V$$

**Figure 7:**
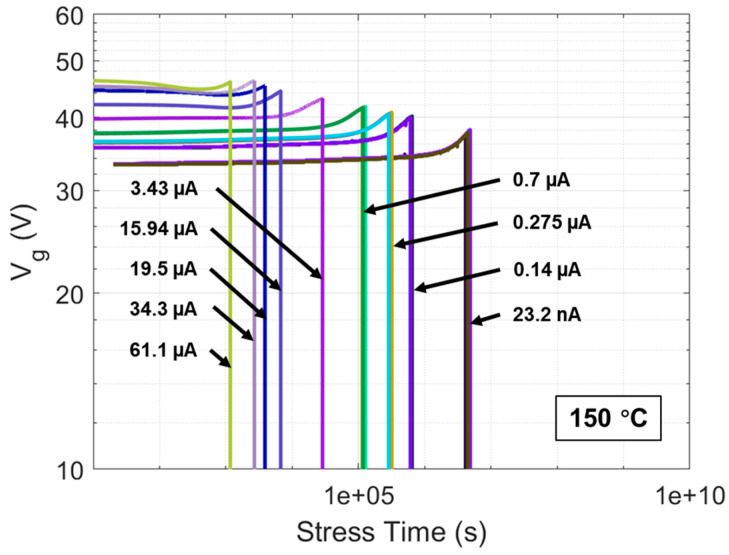
$V_{g}$ curves as a function of stress time until oxide breakdown at 150 °C for multiple DUTs under CCS values of 23.2 nA, 0.14 μA, 0.275 μA, 0.7 μA, 3.43 μA, 15.94 μA, 19.5 μA, 34.3 μA, and 61.1 μA, respectively.

**Figure 8:**
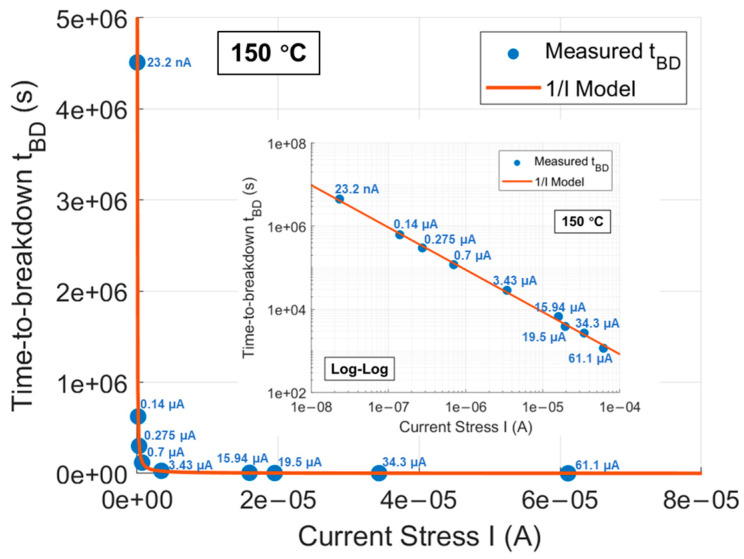
Average $t_{BD}$ of gate oxide at 150 °C under each CCS for multiple DUTs fitted by a 1/I model. The inset shows the log–log scale with a linear relationship.

Figure 7 shows the curves of $V_{g}$ over stress time until oxide breakdown for DUTs under all CCS scenarios used in this work. The applied CCS values include 23.2 nA, 0.14 μA, 0.275 μA, 0.7 μA, 3.43 μA, 15.94 μA, 19.5 μA, 34.3 μA, and 61.1 μA, corresponding to gate oxide electric fields of 7.5, 8, 8.2, 8.5, 9, 9.5, 9.6, 9.8, and 10 MV/cm, respectively, estimated by correlative $V_{g}$ of CCS in Figure 1 divided by the oxide thickness. The $t_{BD}$ of gate oxide in DUTs under each CCS can be extracted when the curves of $V_{g}$ sharply drop and the average $t_{BD}$ at 150 °C under each CCS are reflected in Figure 8. It can be observed that under CCS, $t_{BD}$ follows a 1/I model, which can be expressed as follows:

(14) $$t_{BD}(I)=A\cdot I^{-B}$$

where A and B are constant. For DUTs in this work, under CCS, $t_{BD}(I)$ is fitted by the 1/I model as follows:

(15) $$t_{BD}(I)=\;0.071\;\cdot \;I^{-1.017}\cdot s$$

Or in the log-log scale, Equation (14) can be transformed into the following:

(16) $$\log(t_{BD}\left(I\right))\approx -1.017\;\cdot \;\log\left(I\right)-1.1492$$

Which is in a linear relationship as shown in the inset of Figure 8.

**Figure 9:**
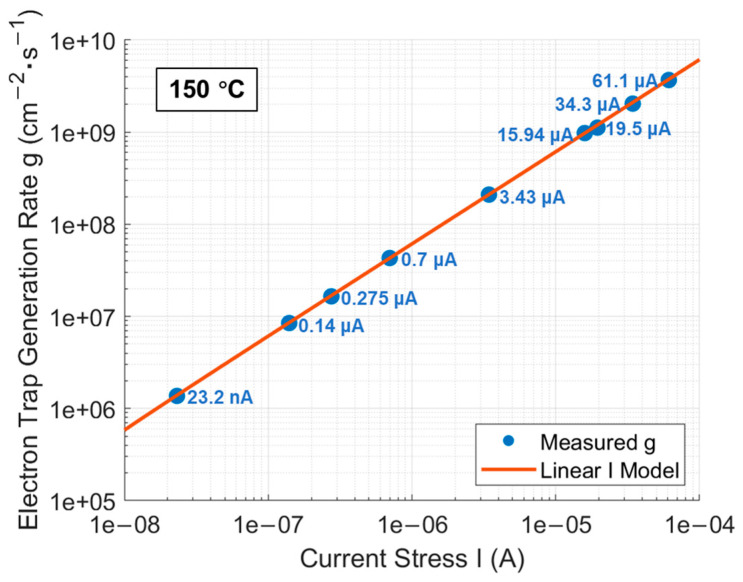
g extracted from the measured data at 150 °C under each CCS fitted by a linear I model.

Using the method described earlier for extracting g of generated electron traps under a specific CCS, g under each CCS is extracted and is presented in Figure 9. It can be observed that g follows a linear I model. The mathematical expression for this linear I model is as follows:

(17) $$g(I)\approx \;6.13\;\times \;10^{13}\;\cdot \;I\;-\;30324.35\;{\mathrm{cm}}^{-2}\;\cdot \;s^{-1}$$

**Figure 10:**
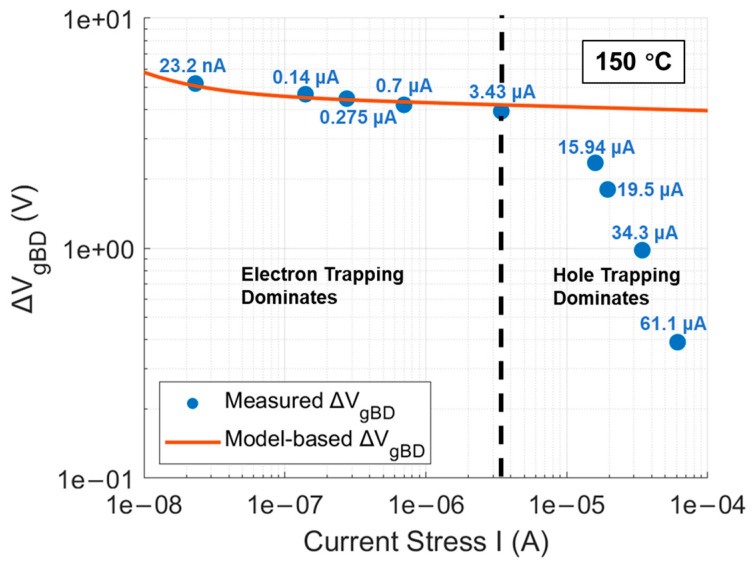
Comparison of model-based $∆V_{gBD}$ with measured $∆V_{gBD}$ at 150 °C.

Therefore, the mathematical expression of $∆V_{gBD}$ as a function of I can be summarized as the combination of Equations (13), (14), and (17). The curve of the mathematical expression is displayed in Figure 10 as model-based $∆V_{gBD}$. Additionally, $∆V_{gBD}$ for DUTs under each CCS can be obtained from Figure 7 by subtracting the initial $V_{g}$ from $V_{g}$ at the point of gate oxide breakdown, which is also reflected in Figure 10. It is observed that the measured $∆V_{gBD}$ under all CCS values not exceeding 3.43 μA highly coincides with the curve of model-based $∆V_{gBD}$ as a function of I. However, as CCS gradually exceeds 3.43 μA, the measured $∆V_{gBD}$ starts to fall below the model expectation. This discrepancy arises because, under CCS not exceeding 3.43 μA, the electron tunneling mechanism is predominantly thermally assisted tunneling (TAT), with the oxide’s trapped charge mainly consisting of electrons, making the electron trapping model applicable in this range. The tunneling electrons lack sufficient energy to trigger enough impact ionization, thus preventing trapped holes induced by anode hole injection (AHI) from dominating over trapped electrons. In contrast, when CCS exceeds 3.43 μA, the electron tunneling mechanism shifts more toward Fowler–Nordheim tunneling (FNT). In this regime, the tunneling electrons possess enough energy at the beginning to cause significant impact ionization, leading to a dominance of trapped holes in the oxide during the first stage of CCS, although trapped electrons subsequently regain dominance. Since the electron trapping model does not account for trapped holes and is solely based on trapped electrons, it is not applicable in the CCS range where trapped holes also play a role. This explanation is corroborated by the trends observed in Figure 7, where under CCS values up to 3.43 μA, the $V_{g}$ curves consistently show an increasing trend due to electron trapping in the oxide throughout the entire stress to breakdown. In contrast, under CCS values exceeding 3.43 μA, the $V_{g}$ curves initially show a decreasing trend due to hole trapping in the oxide, followed by a dominance of electron trapping leading to an increasing trend up to breakdown, and the initial decrease in the $V_{g}$ curves becomes more pronounced as CCS increases beyond 3.43 μA. In summary, it can be concluded that Liang and Hu’s electron trapping model, established for very thin (no more than 10 nm) thermally grown SiO_2_ on Si, is equally applicable to thicker (up to 45 nm in this work) SiO_2_ thermally grown on SiC. This finding will aid in developing a $Q_{BD}$ model for the commercial SiC DUTs.

### 3.2. Modeling of $Q_{BD}$ in Commercial SiC DUTs

**Figure 11:**
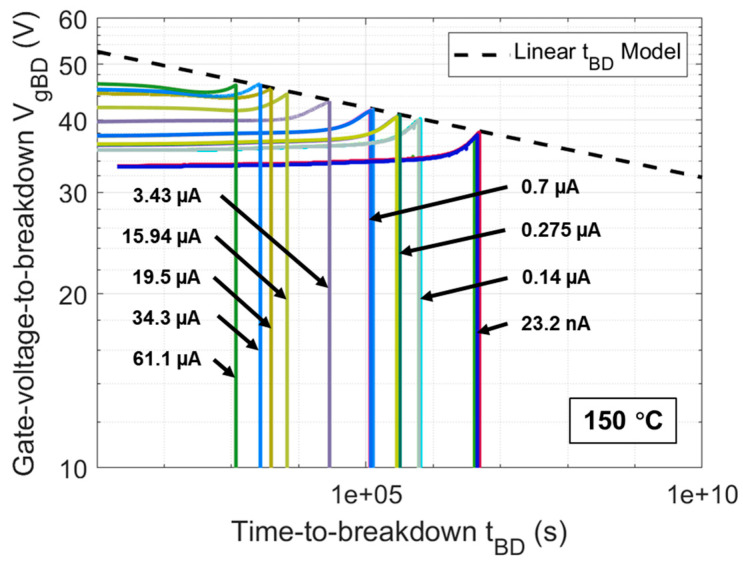
$V_{gBD}$ extracted from $V_{g}$ curves at 150 °C fitted by a linear $t_{BD}$ model.

The $V_{g}$ curves measured in Figure 11 show that the oxide breakdown points of the $V_{g}$ curves under all CCS values follow a linear $t_{BD}$ model on a log–log scale. The mathematical expression for this linear $t_{BD}$ model can be represented as follows:

(18) $$\log V_{gBD}=-0.0242\;\cdot \;\log t_{BD}+1.7448$$

**Figure 12:**
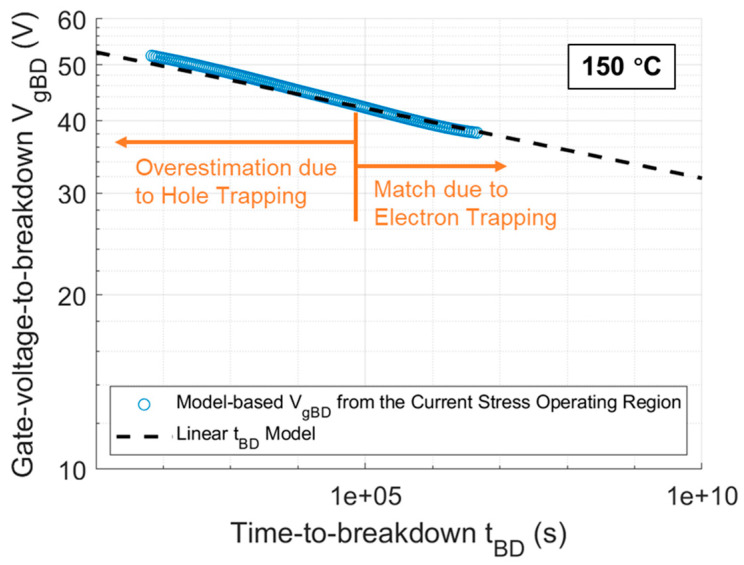
Comparison of model-based $V_{gBD}$ from the current stress operating region at 150 °C with the linear $t_{BD}$ model.

If the segment of the $I_{gss}$ curves for DUTs in Figure 1, ranging from approximately 21 nA to 1.2 mA, is extracted as the current stress operating region, the corresponding $V_{g}$ range is approximately 33 to 48 V. By adding $∆V_{gBD}$, extracted using its mathematical expression from the current stress operating region, to $V_{g}$ corresponding to this region, the $V_{gBD}$ from this region is obtained and then plotted on a log-log scale in Figure 12 for comparison with the linear $t_{BD}$ model from Figure 11 represented by the black dashed line. It is observed that there is a distinct demarcation in the current stress operating region. To the left of this demarcation point, the extracted $V_{gBD}$ is overestimated due to hole trapping, while to the right, the extracted $V_{gBD}$ starts to perfectly match the linear relationship of $V_{gBD}$ measured in DUTs. This strongly validates the feasibility of the mathematical expression for $∆V_{gBD}$ established for the thermally grown gate oxide in commercial SiC DUTs in previous works. It also confirms that $t_{BD}$ under CCS for DUTs, following a 1/I model, is correct and theoretically founded. Therefore, based on Equations (6) and (15), the mathematical model for the $Q_{BD}$ of gate oxide in DUTs under CCS can be established and expressed as follows:

(19) $$Q_{BD}(I)=\;0.071\;\cdot \;I^{-0.017}\cdot C$$

From Equation (19), it can be observed that the power exponent of 1/I is 0.017, which approaches zero, causing the power in the expression to be minimally influenced by I and tending toward 1. Consequently, this makes the $Q_{BD}(I)$ for DUTs approach a constant value of 0.071 C, with the influence of I being almost negligible. This is consistent with the failure mechanism of charge-driven breakdown, theoretically supporting the notion that the failure mechanism of thermally grown SiO_2_ on SiC under CCS is charge-driven breakdown.

### 3.3. Extraction of $Q_{BD}$ in Commercial SiC DUTs under CVS and PVS

**Figure 13:**
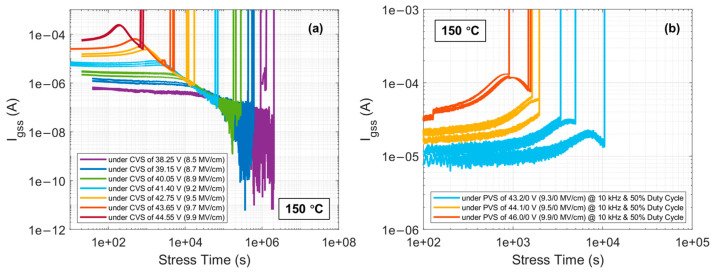
$I_{gss}$ curves as a function of stress time until the oxide breakdown at 150 °C for DUTs under various (**a**) CVS; (**b**) PVS.

**Figure 14:**
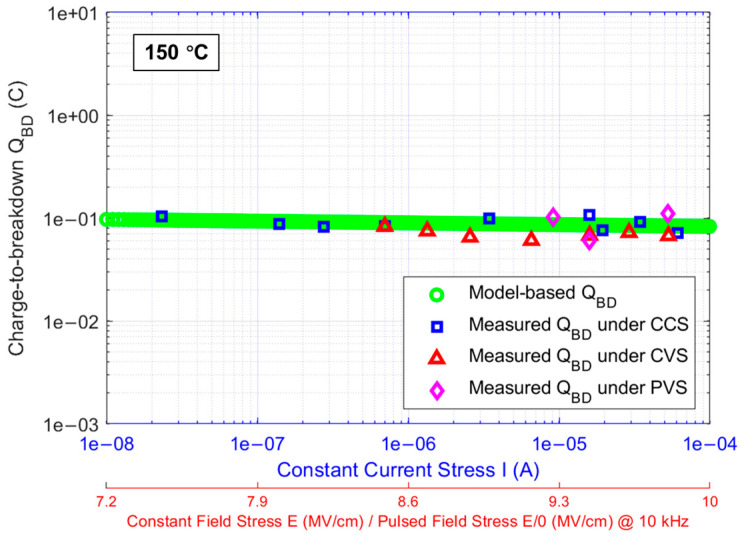
Comparison of model-based $Q_{BD}$ with measured $Q_{BD}$ under CCS, CVS, and PVS at 150 °C.

From Section 3.2, the $Q_{BD}$ model for the gate oxide of DUTs in this work has been established. However, this model has limitations as it is based on the condition of CCS as the stress method for the gate oxide of DUTs. To prove the universality of the model and eliminate the limitations, it is necessary to expand the stress method for the gate oxide of DUTs. CVS, a routine stress method used in the industry for the TDDB test of thermal oxide in commercial SiC power devices, is considered. Additionally, PVS, which more closely replicates the dynamic stress experienced by the thermal oxide in actual operations of commercial SiC power devices, is also taken into account. Figure 13a and Figure 13b respectively show $I_{gss}$ over stress time until the oxide breakdown at 150 °C for DUTs under various CVS and PVS, with different CVS and PVS scenarios also detailed in the figures. Following Equation (6), $Q_{BD}$ values for DUTs under these two stress methods are extracted and presented in Figure 14. As for the $Q_{BD}$ values for DUTs under CCS, they can be easily extracted through the product of constant I and $t_{BD}$, depicted in Figure 14 as well. For comparison, the mathematical-model-based $Q_{BD}$ under CCS is also displayed in Figure 14.

## 4. Discussion

From Figure 14, it can be observed that at 150 °C, CVS and PVS correspond to each other through the electric field stress applied on the gate oxide of DUTs. According to the details in Figure 13 for CVS and PVS scenarios, the difference lies in that under CVS, the gate oxide of DUTs is subjected to a continuous electric field stress until the gate oxide breakdown, whereas under PVS, the same electric field stress applied to the gate oxide of DUTs is a pulsed stress with a frequency of 10 kHz and a duty cycle of 50% until the gate oxide breakdown. The electric field stress applied to the gate oxide is roughly estimated by the ratio of the positive voltage applied to the gate and the gate oxide thickness. Under CCS, the gates of DUTs are subjected to a continuous current stress towards the gate oxide until its breakdown, and CCS corresponds to the electric field stress on the gate oxide under $V_{g}$ associated with the current stress in Figure 1, further corresponding to CVS and PVS. The $Q_{BD}$ values of gate oxide in DUTs extracted under the three different stress methods are distributed in the figure according to the above correspondence and are compared with the model-based $Q_{BD}$ extracted from the $Q_{BD}$ model of thermal gate oxide in DUTs established under CCS. It is significantly observed that the $Q_{BD}$ values of thermal gate oxide in DUTs extracted under the three stress methods conform to the model expectation. The slight differences in the extracted $Q_{BD}$ data fall within the error margin caused by individual differences among the DUTs, which is acceptable and can be almost neglected.

## 5. Conclusions

In this work, the mathematical model established for describing the electron trapping phenomenon in thermal oxide grown on Si, intended for very thin SiO_2_, is considered for transplantation to the gate oxide of commercial SiC power MOSFETs, which is thermally grown on SiC. Given that the mathematical model was initially proven to be applicable only for SiO_2_ grown on Si with a thickness not exceeding 10 nm, its applicability to SiO_2_ thermally grown on SiC, which is approximately 4–5 times thicker in commercial SiC power MOSFETs, is worth discussing. Based on the CCS-TDDB data of the commercial SiC DUTs featuring approximately 45 nm thick sections of thermal gate oxide, the feasibility of this electron trapping model, under conditions where the oxide charge trapping mechanism is predominantly governed by electron trapping, is confirmed in the commercial SiC cases. Following this model, a $Q_{BD}$ model for the thermal gate oxide of commercial SiC DUTs under CCS is established in this work. Apart from the CCS-TDDB test, the CVS-TDDB and PVS-TDDB tests are also conducted on these DUTs. The $Q_{BD}$ values of thermal gate oxide in DUTs are extracted from the TDDB data under the three different stress methods through the integral of $I_{gss}$ over stress time, and are compared with the established $Q_{BD}$ model. The results demonstrate that the measured $Q_{BD}$ values align with the model expectation, indicating that $Q_{BD}$, as a characteristic value of the quality of thermal oxide grown on SiC, remains stable and unaffected by the stressors. This is consistent with and confirms the expectation that the failure mechanism of thermal oxide grown on SiC is charge-driven breakdown. This provides a solid theoretical foundation for establishing a new, more accurate lifetime prediction model based on $Q_{BD}$ for commercial SiC power MOSFETs with thermal gate oxide. Additionally, since $Q_{BD}$ is not affected by the stressors and considering the reduced efficiency in extracting $Q_{BD}$ due to the suppression effect of trapped electrons on $I_{gss}$ under CVS, CCS is recommended as a faster and more accurate method for extracting $Q_{BD}$ in the industry, compared with the conventional CVS, for establishing lifetime prediction models based on $Q_{BD}$ for SiC power MOSFETs with thermal gate oxide.

## Figures and Tables

**Table 1:** General information of DUTs in this work.

Vendor	Voltage Rating (V)	Current Rating (A)	Structure	Est. Oxide Thickness (nm)	Est. Oxide Area (mm^2^)
E	1200	11	Planar	44.15	0.9

## Data Availability

Data are contained within the article.
